# Humanising Government Inquiry: A Realist Examination of a Restorative Approach to Surgical Mesh Harm in Aotearoa New Zealand

**DOI:** 10.1111/hex.70897

**Published:** 2026-10-07

**Authors:** Jo Wailling, Lesley Middleton, Graham Cameron, Carmel Berry

**Affiliations:** ^1^ Te Herenga Waka—Victoria University of Wellington Wellington Aotearoa New Zealand; ^2^ The National Collaborative for Restorative Initiatives in Health Wellington Aotearoa New Zealand; ^3^ Ngāti Ranginui, Waitaha ā Hei, Ngāti Rangiwēwehi, Ngāti Hinerangi Aotearoa New Zealand; ^4^ Mesh Down Under Auckland Aotearoa New Zealand

**Keywords:** government inquiry, moral injury, public inquiry, realist evaluation, restorative approach, restorative responses, surgical mesh

## Abstract

**Background:**

Evidence is limited on how people experience government‐commissioned inquiries, what they value about them, and the impacts of procedures. This study applies realist logic to examine the experiences of those designing and participating in a government‐commissioned restorative approach to harm associated with surgical mesh use in Aotearoa New Zealand. Attention is paid to divergent perspectives about harm and responsibility, desired outcomes, and the multiple identities that shaped people's responses to the programme.

**Methods:**

Using mixed methods, data were collected from documents, interviews, surveys and a research diary to test and refine programme theories. Context‐Mechanism‐Outcome configurations were used to develop theoretical explanations of what worked, for whom, and in what contexts to mitigate, ameliorate, or generate compounded harm.

**Results:**

Compounded harm arises when the different ways individuals make sense of the cognitive, moral, and emotional significance of harm are overlooked. It may also arise when governments and providers do not proactively disclose the inherent risks of healthcare, or the trade‐off between the ideal state and what is possible within constrained resources. Three mechanisms contributed to the restoration of dignity, wellbeing, and trust: collaborative and adaptive restorative design, dignifying storytelling, and responsiveness to the potentially traumatising nature of healthcare harm.

**Discussion:**

Findings underscore the critical importance of acknowledging the prevalence and lived experience of harm, nurturing basic and relational trust, including diverse perspectives, and remaining adaptable if public inquiries are to be effective. Practical recommendations for those commissioning public inquiries emphasise restorative strategies that foster dignity, wellbeing, trust and adaptability while balancing complexity, dynamic human needs, and institutional responsibilities.

**Lived Experience and Public Contribution:**

Patients, families, whānau (extended family), and community representatives were involved in the design of the study, data collection tools, theory testing and refinement, development of the concept of compounded harm, and manuscript preparation.

## 1 Introduction

Government‐commissioned inquiries respond to events of major public concern when officials seek system‐level accountability, reform, and the restoration of public trust [1]. They are increasingly common in the United Kingdom (UK) and colonised countries, where inquiry design is shaped by adversarial traditions, which seek to understand what happened, why, and who is responsible, within a particular legislative and political context. However, evidence relating to inquiry effectiveness and participant experience remains limited. Goodwin et al [2] reviewed fifty‐seven cross‐disciplinary papers on public inquiries and found a significant evidence gap relating to inquiry effectiveness and participant experience. The authors concluded that research must move beyond what can be learned from official reports to explore how people experience inquiries, what they value about them, and the impacts and costs involved.

Inquiries can take many forms. Our contribution focuses specifically on participants' experiences of a Ministry of Health (MOH) commissioned restorative approach to surgical mesh harm in Aotearoa New Zealand (NZ) [3]. Restorative inquiry adapts in response to emerging needs, and we examine what worked, what did not, and under what conditions for the key stakeholders involved.

Surgical mesh is a medical device commonly used to treat pelvic floor disorders and hernias by repairing, reinforcing or supporting weakened tissue. Adverse effects in urogynaecology became a global controversy, prompting government‐commissioned inquiries in several countries [4, 5, 6]. A body of evidence details life‐changing harms; conflicts of interest between consumers, professionals and medical device manufacturers; and epistemic conditions that compounded women's experiences [7, 8].

### 1.1 A Restorative Approach to Surgical Mesh Harm

In 2018, the MOH, with support from Mesh Down Under, surveyed New Zealanders about how they wished to share their experiences. In May 2019, the Office of the Chief Clinical Officers commissioned an unprecedented restorative approach to surgical mesh harm. It represents the first time restorative principles and practices have been applied at scale within a publicly funded health system [3].

A growing movement advocates the use of the restorative approach to account for diverse experiences of harm, health system complexity, and the compounding effects of adversarial responses [9, 10, 11, 12, 13, 14, 15, 16]. Grounded in relational theories of justice, safety and wellbeing [10, 17, 18], restorative responses seek to involve the affected community, consider multiple viewpoints and contributory factors, surface impacts and obligations, and restore wellbeing, dignity and trust [9]. Procedures acknowledge harm, and ask who is hurt, what needs, responsibilities and obligations have arisen, and how harm can be repaired and mitigated in the future [19]. The restorative programme was co‐designed with affected individuals, with relationships at its core. It aimed to listen to citizens' experiences, translate them into action, and evaluate the restorative approach taken [3]. Participants could access dialogical, documentary and support options. Figure 1 outlines the main components.

**Figure 1:**
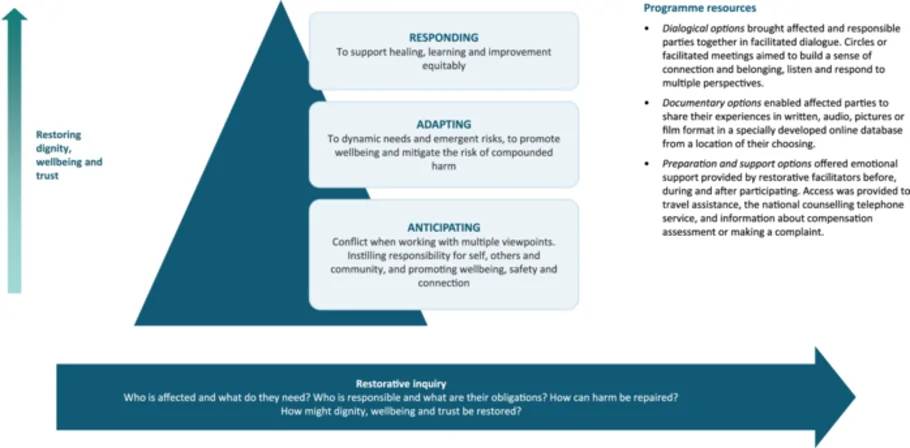
Key components of the surgical mesh programme.

The NZ approach resulted in recommendations aligned with those of other national inquiries, including improving surgical competency, establishing interdisciplinary mesh clinics, and strengthening regulatory oversight of medical devices [4, 5, 6]. Participants in the NZ response also valued learning and healing through mutuality, restoration of dignity and trust, and collaboration for system improvement [18].

### 1.2 Compounded Harm As a Theoretical Lens

Compounded harm is the additional harm generated within interpersonal interactions or procedures that respond to a harmful event or experience and may be associated with distress, mental illness, and suicidal thoughts [9, 11]. It was a key theoretical lens for this study, because the concept was initially defined with harmed consumers during the descriptive evaluation [20]. In health research, compounded harm draws on secondary victimisation scholarship [21, 22]. It is associated with adversarial procedures and practices that negatively affect mental health, access to justice, or system transformation [20, 23, 24]; perceived violations of relational or structural rights, including dignity, consent, and care [17, 18]; and epistemic injustice related to knowledge, sensemaking, and participation [7, 18, 24, 25, 26]. Interdisciplinary teams are increasingly calling for the phenomenon to be recognised and addressed through restorative approaches alongside evidence‐based improvement strategies in global health settings [9, 15, 27, 28].

### 1.3 Realism as a Way of Unpacking Compounded Harm

The descriptive evaluation found the restorative approach worked for most people [20] but provided limited insights into how it worked for different stakeholders or contributed to compounded harm. Realist approaches unpack this ‘black box’ by developing theory‐based explanations of what works, for whom, in which contexts, to what extent, and how [29]. This study examines experiences that illuminate how compounded harm was ameliorated or extended within the context of the restorative response and offers practical guidance for those commissioning inquiries. We focus on three groups: (i) *affected parties* (consumers or clinicians affected by harm), (ii) *responsible parties* (individuals representing a profession or institution with responsibilities related to harm emergence or repair) and (iii) *restorative parties* (people who facilitated face‐to‐face meetings or provided support).

## 2 Research Design

The study applies a realist logic of inquiry to test and refine initial theories using the multilevel and multimethod data collection and analysis required [29, 30]. IF‐THEN statements were used to develop four initial theories about how, and for whom, the restorative programme might work. Theories were informed by the literature and an MOH survey that investigated how people affected by mesh wanted to share their experiences [31]. Table 1 presents the results of the theory‐building stage.

**Table 1:** Initial programme theories.

Systems level	Concepts underlying initial programme theories (key examples)
*Macro‐system* If harm is approached as a human and relational violation, rather than risk to be investigated, then the risk of compounded harm will be mitigated and the human impacts and needs understood.	Theories about how dominant paradigms of safety, harm and justice influence outcomes in the aftermath of a harmful event (preventing harm, learning from harm, reckless and intentional harm, safety science, adversarial and restorative justice) [10, 32, 33, 34, 35]. Feminist, restorative and Indigenous philosophy that focuses on interconnectedness and the relational aspects of justice and wellbeing [17, 36, 37]. Theories on the relational principles of dignity, equity, respect and voluntariness [17, 19, 38, 39]. Scholarship highlighting the challenges of identifying and addressing responsibilities in a complex adaptive system [12, 40, 41].
*Meso‐system* If the community affected by surgical mesh harm are involved in designing the response, then procedural needs will be understood and reflected within the programme design. If restorative programmes develop within a collaborative framework that is representative of the communities providing and receiving healthcare, then individual and collective responsibilities can be identified and responded to in the context of complexity.	Studies and institutional inquiries that identify procedures and practices which fail to address the human and relational impacts or compound the harm experienced [21, 35]. Scholarship highlighting the negative effects of power imbalances and inequity in co‐design and theories promoting community participation and restorative inquiry when co‐creating human‐centred, relational responses [19, 42, 43]. Restorative theory and practices emphasising involvement of the affected community in determining actions for repair and harm mitigation in the future [19, 34].
*Micro‐system* If a restorative response is applied, then substantive and psychological needs will be understood, lived experiences can be safely shared and validated in a safe and supportive environment	Theories about the diversity of justice, safety and wellbeing needs involved in a harmful event, its potentially traumatising nature, and how a continuum of restorative and therapeutic practices might respond [10, 17, 34, 44, 45]. Scholarship identifying multiple perspectives about how risks emerge in health systems, how harm should be responded to, and the role of consumers [46, 47].

### 2.1 Theory Testing and Refinement

In realist research, the Context‐Mechanism‐Outcome (CMO) configuration is the heuristic used to explain how and why interventions generate outcomes [29, 48]. Participants are viewed as ‘theory‐builders’, which helps ensure results identify what mechanisms are firing, what contexts matter, how change is experienced, and the ripple effects [49]. The key output of realist research is middle‐range theory, which is considered highly transferable to other settings [29]. The study therefore applied a participatory approach and an iterative realist approach to theory building and testing using CMOs as the primary unit of analysis. Four data sets were collected to test and refine the theories (see Supporting Information S1: Supporting File 1 for all data sources).

- *Data collected to inform programme design and operations*. Documents and survey data that informed programme design, evaluation tools, and MOH‐commissioned reports. To aid mechanism development, we used Pawson's VICTORE checklist [48] to map the programme architecture (Supporting Information S1: Supporting File 2).
- *Data collected for a descriptive evaluation*. Interviews (*n* = 15) were conducted with a range of participants, including responsible parties (*n* = 9) from public and private institutions and health professions. Affected consumers (*n* = 215) completed a survey (response rate = 50.8%). Demographic patterns were consistent with a national surgical mesh injury claims data set, and respondents were predominantly mesh‐injured NZ European women aged 45–74 [50]. Both data collection tools were informed by participants' hopes and wishes for programme participation (Supporting Information S1: Supporting File 3). Data were reanalysed using realist logic to examine participants' feelings, attitudes and behaviours, with attention to similarities and differences across the three participant groups.
- *Data from the key informant group* responsible for stewardship of restorative initiatives across the health system. Eight participants contributed to realist interviews (Supporting Information S1: Supporting File 3). Five women and three men represented five government bodies, one provider, and two consumer advocates. Two were Māori with restorative justice experience, one was new to healthcare, and the others had mainly applied restorative approaches in healthcare.
- *Data captured in a research diary*. JW was embedded in the Ministry inquiry from the outset and recorded observations relating to the different experiences and espoused rationale for decision‐making of the three participant groups.

The process of theory development and refinement is illustrated in Figure 2 (see Supporting Information S1: Supporting File 4 for additional detail).

**Figure 2:**
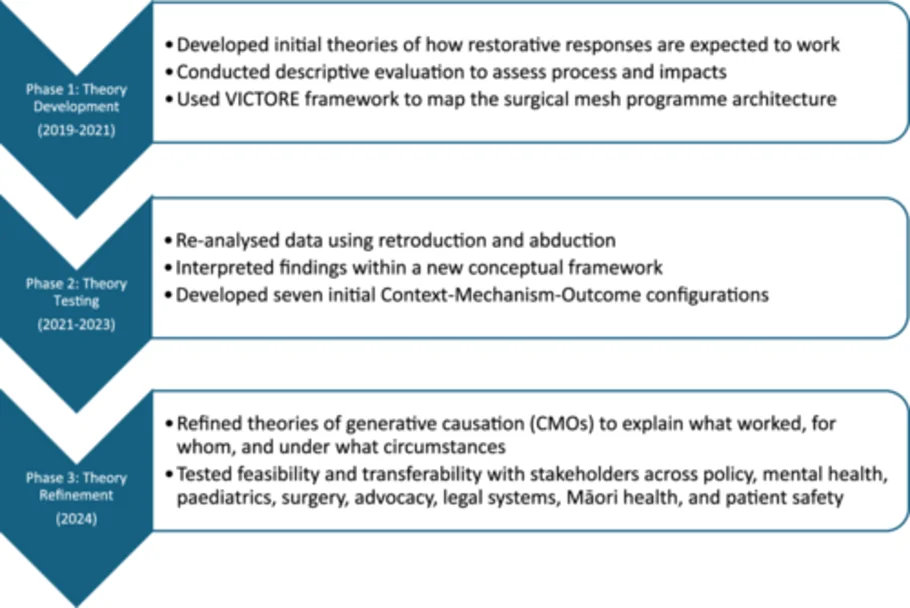
Theory testing and refinement.

## 3 Results

Study participants are detailed in Table 2. We identify three contexts in which participants' experiences of the restorative programme differed:

- Divergent perspectives about harm and who is responsible for addressing it.
- Different outcomes that responsible and affected parties desired from the restorative programme.
- Lived experience and the multiplicity of identities that shaped people's responses to the programme.

**Table 2:** Study participants.

Participant Category	Definition	Subcategory	Theory Testing (ID number)	Theory Refinement (ID number)	Examples
Affected party	An individual who has experienced healthcare harm and identifies as a consumer	Consumer (C)	215 survey participants (C) 3 interview participants (IP)	n/a	Women and men who had undergone mesh‐related surgery or their partner, spouse, family or whānau of someone harmed, consumer advocates
Affected party	An individual who has experienced surgical mesh harm who identifies as a healthcare professional	Affected health professional (AHP)	6 survey participants (AHP)	n/a	Surgeons, general practitioners, nurses, allied health professionals
Responsible party	A healthcare professional who is perceived to be actually or potentially responsible for: (a) the conditions that contributed to surgical mesh harm or (b) acting for repair and prevention in the aftermath of surgical mesh harm; (c) developing policy that mitigates or responds to harm in the future	Responsible party (RP)	9 interview participants (IP)	n/a	Representatives of private hospitals, responding institutions, regulators, professional bodies, and the government MOH, clinicians (general, urology and obstetric surgeons; psychiatrists)
Restorative party	A person with qualifications and experience in facilitating restorative processes	n/a	3 interview participants (IP)	n/a	Social workers Restorative practitioners
Workshop participant	An individual responsible for guiding the development of restorative policy and practice in NZ.	n/a	n/a	8 (WP)	Consumer advocate Clinician Policy maker Legal professional Māori leader

### 3.1 Divergent Perspectives of Harm and Responsibility

Mesh use was depicted as a dehumanising violation of women that subjugated them to research or personal gain. Drawing parallels with The Unfortunate Experiment,^1^ affected consumers frequently expressed the view that history was repeating or referred to themselves as ‘*guinea pigs*’ (e.g., C61, C101, C129):

These perspectives stressed that women with surgical mesh complications have not been treated with respect, they have not been treated fairly, they have not been treated with dignity. They have not been communicated with, they have been lied to and coerced, they have been part of a massive uncontrolled experiment, and their complaints have not been given a fair hearing [3].

Consumers typically blamed doctors or mesh manufacturers, describing consent processes as coercive and injuries as the result of intentional or careless acts. They rarely accepted that ‘the system’ was to blame, wanted someone to take responsibility, and distinguished mesh injury from the institutional procedures and interactions that followed:

The physical injury is very serious, but these things happen and can be tolerated. With the right help and treatment, it is possible to address. Far, far worse for me has been the denial of two specialists who adamantly refused to even consider that I had any kind of injury.

(C89)

Consumers expected the system to provide compensation and uphold their rights. Most shared that they felt betrayed by the medical professions and by two institutions: the Accident Compensation Corporation, which assesses and provides treatment injury compensation [51]; and the Health and Disability Commissioner, which upholds the Code of Health and Disability Consumers' Rights [52, 53]. Consumers cannot pursue civil litigation in NZ, and responsible parties who were clinicians emphasised that these protections matter, because risks cannot be eliminated:

Whatever we do to people has a risk of harm. The only safe operation is the one we don't do because it can't go wrong.

(IP10)

Most responsible parties saw harm as emergent, shaped by factors they could not control, and indicated that taking responsibility for ‘the system’ was distressing. Because mesh use occurred largely in private hospitals, some were angered that public servants and taxpayers were left carrying the burden (RD76, RD112). Others were surprised to discover that policy and practice underpinned by adversarial principles were not working as intended, and had constrained their willingness or ability to respond in ways consumers expected:

A lot of [responsible parties] …expected things to be working in a certain way and were a bit horrified to find out that their members or that their policies in action weren't having the effect that they had intended or hoped for.

(IP2)

During theory refinement, workshop participants described harm and responsibility as distributed across collective actions. Recent political shifts were seen to constrain public servants' ability to act on programme recommendations, reigniting conflict with consumers. When responsibilities remained unaddressed, both adversarial and restorative approaches were associated with ‘*the fallacy of system change’* (W6). The fallacy referred to identifying issues or solutions without action, or making recommendations deemed unfeasible or too resource‐intensive.

### 3.2 Multiplicity, Moral Injury and Shame

Three identities emerged as participants reflected on how the programme worked: procedural roles (e.g., victim, responsible party), social identities derived from group membership (e.g., advocate, mother, healer), and cultural identities shaped by customs, values and worldviews (e.g., Māori, clinician, public servant). Most people held more than one identity (multiplicity). Incongruence between chosen and ascribed identities was described as harmful and often accompanied by shame, guilt, anger, or a sense of moral imbalance between parties.

All parties provided accounts that featured elements of moral injury. Moral injury is grounded in consciousness when people are unable to make sense of experiences that challenge their identities, moral codes, or ability to trust themselves or others [54]. Responsible parties often felt betrayed by ‘the system’ or by colleagues as they struggled to make sense of the devastating consequences of harm while discharging their professional duties:

I ran into trouble, because sometimes I would try to be [a person] …but I could never not be a public servant supporting a Minister.

(IP4)

Consumers' stories often portrayed responsible parties as violating ethical or moral standards. Surgeons were described as selfish, paternalistic and financially driven members of an ‘*old boys' network*’ (C133), challenging doctors' identities as competent, patient‐centred healers acting in accordance with professional practice:

I guess the fear is – how is the profession going to look? We like to think that we look caring and kind and competent. There was a very real risk that we would look uncaring, dismissing people's problems, unkind and incompetent, because we were doing something that was now being shown to be associated with far more harm than had been realised.

(IP10)

A key point of controversy was the suggestion that consumers were victims of ‘*medical rape*’ and several women shared responses that triggered distressing memories [3]. The term was associated with consent processes, the living experience of mesh injury, claims assessment procedures, and was accompanied by expressions of shame or moral outrage:

Not long after my second mesh removal operation, I had to have an ultrasound. The radiographer violated me so appallingly that I felt I had been raped. In desperation I went to my GP, I was an emotional wreck in severe pain. The GP did f*** all about it. I have no trust in the system.

(C117)

Responsible parties usually disregarded the term rape as emotive and shaming (RD14). Public shaming was a key concern influencing their choice to participate and was associated with not living up to social norms, feeling personally responsible for the actions of others, and professional identity. During theory refinement, workshop attendees identified that the potential for retributive or shaming acts by the coroner, or the media, were barriers to expressing remorse.

The most prevalent consumer complaint was being told that symptoms were ‘*in their head*’ (C79) and was often accompanied by reports of gaslighting and betrayal. Epistemic injustice, coupled with mesh injury, was also associated with the loss of identities tied to masculinity or femininity, such as being a parent, supportive partner, desirable lover, or competent professional. Desired identities were replaced by unwanted stereotypes—ascribed by doctors, complaint reviewers, or claims assessors—linked to credibility deficits such as the ‘hysterical woman’. Many consumers associated this identity loss with mental illness, post‐traumatic stress, and suicidality.

Many consumers identified with victimhood, associating the identity with violations of consent or bodily autonomy, while others saw themselves as survivors. Most affected health professionals considered the absorption of harm to be a professional or moral responsibility or rejected the notion that they were victims (RD70, RD82). They reported that persistent bullying, whistleblowing, or witnessing suffering led to significant impacts on their wellbeing, including helplessness, powerlessness, and burnout, but described their distress as minor in comparison with that of consumers.

### 3.3 Divergent Perspectives of Outcomes Desired From the Restorative Approach

The conflict between affected and responsible parties was more extensive and complex than restorative parties had anticipated. Divergent perspectives and competing needs had generated adversarial interpersonal conflicts nested within broader institutional and societal settings. Interview data were saturated with descriptions of fighting, toxicity, incivility, drama, and disrespect between consumers and responsible parties (IP5, IP6, IP12, IP13, IP10). These dynamics shaped expectations about what the programme could realistically achieve.

Consumers wanted processes that acknowledged their experiences, offered support or compensation, and enabled them to alert the public about risks. They stressed, ‘*it was time for the mesh‐injured to be heard*' (IP13) and expected informed choices about how and with whom they shared their experiences. Responsible parties agreed, but also valued timely, collaborative responses from involved institutions and distinguished the restorative approach from one that sympathised with the plight of consumers or perpetuated powerlessness:

None of those people want sympathy. They want you to understand them, they want to help themselves and they want help where it's available. They don't want the ‘poor you’ kind of thing. That doesn't reduce distress, and it doesn't help them in any regard.

(IP11)

Both parties preferred an approach that enhanced system safety and provided restorative responses to the human impacts. During theory refinement, the restoration of wellbeing, dignity, and trust was depicted as a unique or therapeutic outcome of restorative responses, particularly when contrasted with adversarial systems:

There is the legalistic reductionistic mandated process that is prescribed and followed versus the restorative approach which is relational and aimed at addressing harms and restoring people's trust and dignity for healing…. One is about addressing the needs of the people. The other, the needs of the system.

(WP6)

In realist terms, a mechanism is not simply a process or activity but a generative explanation of how outcomes occur [29]. In the following section, we apply Dalkin and colleagues' definition of a mechanism [55]. We examine the *resources* provided by the restorative programme and how individuals or groups responded, meaning their *reasoning* through interpretation, decision‐making, motivation, and action. The three key mechanisms that mitigated or ameliorated compounded harm are presented in Table 3.

**Table 3:** Mechanisms.

Mechanism	Resources and strategies	Response
Collaborative, facilitated design	Skilled facilitator(s) attentive to power imbalances and invested in the rights and needs of all parties. Relational ground rules are supported and maintained by the facilitator and wider group. Restorative inquiry underpins interpersonal interactions, procedures and adaptations.	Equity of voice in programme co‐design and the impacts, obligations and outcomes desired. Feeling safe and supported during conflict and collaborating for shared purpose. Relational ‘slack’ enables adaptive capacity while maintaining focus on restorative goals.
Dignified storytelling	Participants can make an informed choice about the potential impacts of storytelling and share experiences how, when, and with whom they choose. Multiple perspectives of harm and living experience are witnessed and validated by loved ones, peers and/or responsible parties. Māori lead restorative approaches for iwi, hapū, and whānau.	Dignifying mutuality, epistemic agency, and renewed sense of hope about the future; in ways that restore basic trust and desired identities. Shame, guilt, anger and other emotions can be safely expressed. Culturally safe expression.
Trauma‐responsive support	Access to a range of resources adapted in response to the emergent needs of individuals (cultural, psychological, emotional, relational, physical). Emotional support provided by the communities that individuals identify with (e.g., peers, experts by experience). Access to formal mental health support.	The agency to determine what support is required to restore or maintain wellbeing, make sense of harmful experiences individually and/or collectively, and safely engage with procedures and practices (or not)

### 3.4 Collaborative, Facilitated Design

Skilled facilitators adopted an adaptive co‐design philosophy grounded in restorative inquiry. This strategy sought to distribute decision‐making, respond to participants' changing needs within available resources, and develop relational ‘slack’. Interviews indicated that collaborative design and skilled facilitation fostered equity, connection, and epistemic agency, and served as ‘*a third party to hold the mirror*’ (IP13). The restorative inquiry framework was embedded consistently across interactions, procedures, and evaluation design:

We changed lots of things. All seem so subtle, [but] we were really intentional about [it]…After every [Listening Circle], we would look at the questions again or would highlight, ‘oh, when you said this, I think this had this impact’ or ‘could you say it like this differently’ or ‘let's not do it that way ever, ever again’.

(IP2)

Consistent application of restorative inquiry, coupled with adaptations in response to feedback, was highlighted as a key programme strength by all parties (IP1, IP2, IP6, IP12).

At the outset of each face‐to‐face meeting, facilitators sought agreement to a set of ground rules: listening and speaking respectfully, sharing time fairly, remaining mindful of others' experiences, and maintaining confidentiality. This shared commitment contributed to participants' sense of safety because they were ‘*mindful of boundaries*’ (C50); ‘*everyone was allowed to speak, and everyone was supported*’ (C33); and these ‘*rights provided a clear definition of process and how our presentation would be handled*’ (C8).

Responsible parties shared the view that an intermediary role had been essential for success but placed more weight on neutrality:

One of the risks is that [some restorative parties] became advocates at one stage rather than that neutral facilitator. It was mainly commentary around what would be the solutions that the participants would be looking for or outcomes for staff.

(IP6)

Restorative parties said that humans could not be simultaneously empathic, compassionate, and neutral. They described their role as multi‐partial and valued upholding equity and distributing power.

During theory refinement, workshop attendees noted that while independent reviews are often viewed positively, they compound harm if relational aspects are neglected. The group favoured responses that promote agency and challenged power imbalances, especially when one party felt unsafe due to perceived bias, culturally unsafe practices, lack of transparency, or restricted access to information.

### 3.5 Dignified Storytelling

Providing multiple storytelling options aimed to promote testimonial justice, dignity, and epistemic agency. Affected parties could choose from dialogical options (Circles and face‐to‐face meetings) or documentary options (written, vlog, or visual) to share their experiences (Figure 1). Uptake of Listening Circles was higher than predicted (*n* = 260 *vs. n* = 141), although consumers most often chose the story database option (*n* = 485).

Multiple survey comments indicate that writing about experiences was cathartic, allowed adequate time for reflection, and ensured access to family or whānau support. Many consumers (*n* = 140) accessed more than one storytelling option or shared artefacts (e.g., pictures of mutilated bodies, extracted mesh, artwork). Several individuals indicated that restricted access to private meetings, or participation in Circles that included men, compounded harm.

Most consumers reported feeling dignified by peers and responsible parties in Circles which were associated with sensemaking, validation or mutual healing and learning:

Because I saw the attention given to each mesh‐injured person's story, I felt confident to share openly when it came to my turn…The most powerful example of ‘being heard’ was when one lady told her tragic story and the Chief Nurse had tears.

(C55)

The Circle I attended had it all from defiant hand‐slamming, to barely able to move to showdowns with ACC, to several surgeries for repair work, to doubting doctors, to life‐altering outcomes. I feel lucky to have been able to hear the stories of other women and to be able to put my experience into perspective.

(C182)

The MOH‐commissioned report [3] was referenced as a dignity tool because it publicly validated consumers' experiences. Ninety statements about feeling hopeful appeared in the evaluation survey alongside comments related to validation of lived experience and chosen identities. Consumers associated dignity with the restoration of trust and renewed confidence that action would follow:

I did feel heard, and once again, I felt hope that changes would be made to help mesh‐injured New Zealanders.

(C59)

Most responsible parties shared that listening without responding defensively enabled them to connect emotionally and intellectually to stories, which provided powerful motivation for collaborative action. Circles were referenced as safe spaces to be human, rather than being bound to a professional identity, and to experience mutually beneficial outcomes:

I do think there was more reciprocity and understanding, mutual benefit and more openness. Because of the people in [Circles] being more open, then that encouraged openness in others as well.

(IP8)

Occasionally, responsible parties encountered personally directed expressions of anger, blame, or threats of violence. Such experiences were distressing, even when facilitators intervened:

[Consumers] are using the excuse ‘It's not about you, it's about patients in general’ so they can depersonalise the person they're being vitriolic to, which doesn't depersonalise it while you're listening to them. That was really hard. You're sort of sitting there, sort of being hated.

(IP6)

During theory refinement, workshop attendees agreed that face‐to‐face encounters support dignifying mutuality and ameliorate harm, but emphasised voluntary, informed participation. They affirmed that epistemic agency is essential in the aftermath of harm, and that declining a face‐to‐face meeting can also be empowering:

The worst [events], I wasn't strong enough to be in the room, I needed to write down our experiences… I didn't have enough power to be present. My trust wasn't there ‐ that I would be treated well ‐ and writing it down makes it permanent. I had more power.

(WP5)

Responsible parties associated the adversarial system with forced participation that had laid a legacy of guardedness and mistrust:

[Responsible parties] may say at the time, ‘Yes, I should not have done that, done it differently, or that was a mistake…’. Even if that is confidential… If you are then sitting in a Coroner's Court, and someone is cross‐examining you, you can't take a different position.

(WP2)

Māori participants associated the adversarial system with colonial expression. For Māori, restoring ‘*mana tangata*' (WP4)—the power and status accrued through one's leadership talents, human rights, and the dignity of people—required iwi, hapū, and whānau to own and lead culturally safe restorative approaches. Cultural safety requires that power imbalances be acknowledged, historical context addressed, and cultural identity be expressed safely without fear of discrimination or bias [56].

### 3.6 Trauma‐Responsive Support

Many elements of the surgical mesh programme were intended to reduce distress. Participants could choose from a range of formal supports, co‐designed with mesh‐injured people, to foster agency before, during, and after participation (Figure 1). Emotional support provided by restorative facilitators was intended to convey care, reassurance, empathy, comfort, and acceptance. Information was also provided about access to the national counselling line and to complaint or compensation processes.

Many consumers had concealed suicidality from loved ones and related Circle participation to seeking support or sharing their experience with family for the first time. The community setting was depicted as the key source of emotional support the programme provided:

What was fantastic and unexpected was the support from the other members attending the Circle. I realise now that they have been there, done that (or had it done to them) and their empathy was just amazing. I am so glad that they were there.

(C66)

Survey comments indicate consumers were aware of the options available, but few accessed them. Those who did were more distressed than expected and did not appreciate being referred to institutions perceived to have compounded harm. Several desired intensive support, psychological care or specialist services for addiction or mental illness and were distressed that tailored options were unavailable:

The idea is good, but there should have been personal follow‐up, not only after the session, but in the weeks following. Many of us have been suffering for years, and this opened a can of emotions that had been trapped… The triggers went on for months.

(C39)

Responsible and restorative parties usually viewed witnessing human suffering as a professional duty or skill. They relied heavily on peer support, although restorative parties also arranged professional supervision and counselling. Attending multiple face‐to‐face meetings, the severity of harm, operational pressures, nested conflict, and other workload commitments had a greater impact than anticipated:

That first round [of Listening Circles] was just too much, was simply unsustainable and…I don't want to use the word dangerous, but I personally felt the closest to professional burnout that I have ever been.

(IP3)

A relationship was reported between exposure to suffering and compounded harm. Important operational adaptations included recruiting additional parties during the listening phase and using the restorative inquiry framework to explore emotions and moral impacts.

During theory refinement, workshop participants suggested that any adversarial or restorative approach that places sustained burdens on a small number of individuals may be traumatising. They observed that exposure to potentially traumatic events is common in healthcare settings and across NZ society, shaping experience at individual, collective, and intergenerational levels. Examples included clinicians' routine exposure to death, witnessing the suffering of loved ones during treatment, and the intergenerational effects of colonisation. Participants therefore emphasised the need for dynamic, responsive support options and community support capable of addressing human and cultural complexity:

Support‐based resources are critical. It's cultural safety, it's emotional safety, it's relational safety, having an awareness that people have experienced a traumatic event, and interactions need to be recognised in a way that supports that.

(WP4)

### 3.7 Summary

This section applied a realist logic to explain how, for whom, and in what circumstances restorative design, storytelling practices, and emotional support worked—or did not work—to mitigate, ameliorate, or generate compounded harm across three participant groups. Three mechanisms were identified (Table 3): adaptive restorative design, dignified storytelling, and responsiveness to the potentially traumatising nature of healthcare harm. In specific contexts, compounded harm could still be generated:

- If responsible or restorative parties bear witness to suffering for intense or prolonged periods, they can experience moral injury or distress.
- If restorative or responsible parties lead a process in which recommendations are not supported because of political will, or the resource or legislative efforts required, they may experience learned powerlessness, moral injury or distress.
- If affected consumers' substantive needs remain unmet (e.g., psychological support, compensation, mesh removal surgery) due to how ‘no fault, no blame’ treatment injury legislation is enacted, the response can be viewed as unjust and uncaring by representatives across party groups. For background, the Accident Compensation Act 2001 [51] removes consumers' right to sue health providers or mesh manufacturers. They are not acknowledged as victims, which is important because victims of crime can access government‐funded psychological, cultural and restorative justice support [57, 58].
- If affected consumers do not feel informed or are unable to access support tailored to their needs, they may experience epistemic injustice, distress, or illness.
- If governments or healthcare providers do not proactively disclose the inherently risky nature of healthcare, the trade‐off between the ideal state (system safety) and what is possible within constrained resources (the reality), and the evidence base for recommendations, inquiries will be associated with the ‘fallacy of system change’.

## 4 Discussion

Inquiries aspire to procedural fairness but frequently fall short in ways that shape participants' experience, public trust and access to justice [2]. Our study contributes to a significant knowledge gap about how people experience them, what they value, and the impacts involved. Government‐commissioned inquiries aim to restore public trust in institutions. The literature suggests that health systems can either generate or undermine trust through transparency, responsiveness, safety, fairness, epistemic agency and alignment with patient interests [7, 15, 59]. Findings shed light on how the restoration of trust is conceptualised and understood by different parties and indicate that restoring public trust is a complex, long‐term endeavour that requires foundational work. For consumers, restoring *basic trust*—the ability to trust oneself and others—is essential for self‐esteem, healthy relationships, and recovery from trauma [45, 60]. Responsible and affected parties also sought *relational trust*, which Weber and Carter [61] define as the belief that the other will take one's perspective into account and refrain from acting in ways that violate moral standards.

Results illuminate the complexity of the conflict that influenced programme implementation. The conflict fulfils Dugan's (1996) criteria for nested conflict because it extends across relational, structural and system levels [62]. Conflicts between surgeons and consumers were embedded within broader tensions between advocates and government, and were shaped by interdependent structures such as regulation, inequity, and bias. A major source of relational conflict was the tendency of responsible and affected parties to assign each other stigmatising or harmful identities. Mistrust was intensified by multiple procedural, social, professional, and cultural identities. The restorative programme sought to address these dynamics through honest, empathic facilitation. The approach recognised harm, responded to individual and collective needs, and attended to underlying power relations. Our findings suggest that once relational trust is tentatively rebuilt, restoring public trust requires rigorous system improvement informed by differing views of harm and responsibility.

Participants' expressions of moral outrage were particularly striking and are consistent with research on the human experience of violence [63]. Moral anger arises from perceived intent to harm, whereas actual harm tends to elicit empathy for victims [64]. Responsible parties linked this anger to the actions of private providers and manufacturers that compelled them to assume responsibility by proxy, whereas consumers generally believed that mesh harm resulted from reckless, intentional, or careless acts. They insisted that someone should be held accountable and rejected explanations that attributed responsibility solely to ‘the system’. These findings matter for three reasons. First, perceived intentionality increases the likelihood of post‐traumatic stress over time [65]. Second, both groups described psychological and moral harms that affected basic integrity and the high degree of trust expected in healthcare. Third, contemporary definitions of moral injury align with the complex individual, interpersonal, and institutional harms surfaced in the study [66].

Responsible parties viewed harm as an emergent outcome shaped by multiple factors. This perspective aligns with safety science [10, 33, 46] and appears in NZ health policy [67], although the inherent risks of healthcare are rarely discussed publicly. Literature also points to a global commitment to ‘zero harm’ as a ‘*sacred core value*’ of healthcare institutions [68]. Our findings suggest that the belief that harm is always preventable can itself be harmful. It shapes expectations of perfect safety, influences treatment decisions, minimises the lived experience of harm, and raises expectations that harm can be swiftly remedied.

Adversarial procedures assume truth emerges when two opposing sides present competing narratives [69]. Our findings reveal that the dignified storytelling provided by a restorative approach was beneficial for most people. Research in mental health settings also concludes that storytelling, mutuality, and bearing witness can be therapeutic, support reconnection, sensemaking, reframe violent experiences, and rebuild moral frameworks [70, 71, 72]. Storytelling is also vital for healing in Indigenous cultures [73], though it may not always be cathartic. The finding that repeated exposure to suffering or shame can generate compounded harm for responsible and restorative parties is consistent with research examining other official inquiries. Wyles et al. [74] concluded that bearing witness can increase distress—also noting it hinders forgiveness for some individuals.

Restorative scholars suggest that state‐led programmes can limit restorative potential by setting parameters for oversight, delivery capacity, referral pathways, and evaluation criteria [75]. The NZ approach produced similar recommendations to adversarial public inquiries elsewhere, often considered weak in the hierarchy of improvement effectiveness because they target individual behaviours rather than systemic or structural action. Government institutions and professionals still determine which recommendations are prioritised, who designs and implements actions, how they are delivered, and the timeframes involved [76]. A shift in political ideology has halted the implementation of actions related to therapeutic products legislation, and a public apology has not yet been provided. However, most recommendations have progressed or been delivered. The programme also sparked collaborative national efforts within a Treaty of Waitangi framework, with the restorative approach now embedded in national policy, standards, and the Mental Health Act 2026 [67, 77, 78].

### 4.1 Implications

A restorative approach offers important advantages, but the benefits are not universal. The key contribution of the study is the development of middle‐range theories that explain how a restorative approach ameliorated, mitigated or compounded harm for different people. Our findings suggest that those commissioning, delivering, or evaluating an inquiry should focus on four areas: collaborative design, multiple storytelling options underpinned by restorative inquiry, trauma‐responsive supports, and transparency alongside responsibility for harm and repair. Early adopters should anticipate a range of ripple effects. Compounded harm is more likely when institutional or political priorities take precedence over design quality or fail to support informed choice and human agency. In practical terms, the justice needs of affected and responsible parties should be surfaced, clarified, and treated as the key evidence base for inquiry design. Embedding researchers in the end‐to‐end process enables a nuanced understanding of what works (or not) for different groups.

Harm creates a moral imbalance between the people who are viewed as responsible for the conditions that enable harm and those affected by it. Public inquiries are typically led by experts valued for technical expertise, objectivity, or procedural adherence. Yet opportunities to cultivate basic and relational trust are often overlooked, despite their importance for mutuality. When responses fail to engage with differing or competing stakeholder interests, including those of government institutions, adversarial dynamics can emerge. These dynamics may constrain restorative outcomes, effective resolution, and meaningful progress. A collaborative framework informed by diverse perspectives and storytelling practices is better able to accommodate the cultural and cognitive diversity required for recovery, knowledge sharing, and improvement. Facilitating divergent viewpoints and respectful dialogue while remaining attentive to the needs of all participants requires substantial skill. Restorative inquiry can account for diverse viewpoints and is responsive to the needs of people and institutions. It should therefore be continuously integrated into procedures and interactions to build the adaptive capacity required for community safety and restoration.

Results illuminate that healthcare harm is potentially traumatising and is grounded in the context of individuals and the systems that respond. Findings are particularly relevant for addressing harm experienced by women, with the NZ approach offering insights for maternity inquiries [28]. Like mesh‐related harm, maternity harm has been viewed as both a form of dehumanisation and a human rights issue [79]. Although clinicians have not welcomed this framing, systemic problems and nested conflict persist despite multiple inquiries. Trauma's cumulative effects on mental health increase with the severity and frequency of harmful experiences [80]. Health systems must build trauma‐responsive frameworks to mitigate compounded harm. Inquiry frameworks must prioritise support for all involved through moral, peer, emotional, cultural, and interdisciplinary assistance.

Complexity can hinder restorative efforts, especially when views of harm and responsibility differ. Responsibility is shaped by individuals, groups, and broader systems that influence who achieves desired outcomes. New Zealanders harmed by surgical mesh have unmet needs, while responsible parties face challenges from changing policies, political ideology, and prevailing safety norms. If institutions want to nurture restorative potential, a collaborative interdisciplinary approach, grounded in cultural safety and allyship with experts by experience, is essential [9, 77, 81]. Participants reported that the Accident Compensation Act 2001 shields private providers and limits the Government's ability to seek redress from overseas manufacturers and providers. Investigating this assertion may benefit the public purse and help fund responsive services. Policymakers could also consider extending legislative protections to recognise harmed consumers as victim‐survivors. This would give consumers access to support and restorative justice more closely aligned with that offered to victim‐survivors of violence.

### 4.2 Strengths and Limitations

This study was not designed to determine whether a restorative approach is more effective than government or judicial inquiries, or whether the benefits identified endure over time. Mitigating the human and economic impacts of health policy and practice is a global priority [82], and more research is needed to understand how inquiries work and whose needs they address. Further investigation of compounded harm is also needed to inform responsive policy and clarify how therapeutic interventions should respond to the cognitive, moral, and emotional meanings people assign to harmful events.

Studies of social reality are limited in the claims they can make about generalisability. Realist researchers may privilege theories aligned with their own worldviews, and participants' interpretations during theory refinement may be critiqued as subjective and influenced by researcher reflections [29]. Those limitations were mitigated by several strengths: a participatory approach; theory as the unit of analysis; multiple levels of data collection, analysis, and triangulation; continuous informal testing with diverse stakeholders over time; and theory refinement with stakeholders who were not involved in the programme. Findings are likely to be particularly transferable within NZ and to inquiries addressing harm experienced by women or arising in surgical or mental health settings. Relevance for Māori will ultimately be determined by iwi (tribes), hapū (sub‐tribes), and whānau (extended family).

## 5 Conclusion

This study shows that a restorative approach to government inquiry can mitigate compounded harm through collaborative design, dignifying storytelling, and trauma‐responsive support. To be effective, such inquiries must engage diverse perspectives, attend to power, recognise nested conflict, and adapt to complexity. Adversarial systems and unmet justice needs can themselves compound harm, underscoring the importance of a human‐centred approach. Future inquiries should prioritise the restoration of basic and relational trust and pair this with an evidence‐based approach to safety improvement.

### 5.1 Address Where Research Was Undertaken

School of Health Te Herenga Waka‐Victoria University of Wellington, 45 Kelburn Parade, Wellington 6012, Aotearoa New Zealand.

## Author Contributions

**Jo Wailling:** conceptualisation, investigation, funding acquisition, writing – original draft, methodology, writing – review and editing, formal analysis. **Lesley Middleton:** conceptualisation, writing – review and editing. **Graham Cameron:** writing – review and editing, formal analysis. **Carmel Berry:** writing – review and editing.

## Ethics Statement

The study was approved by the Victoria University of Wellington Central Human Ethics Committee (27693 & 28500). Because research in colonised societies must acknowledge the impacts of colonisation, Te Tiriti o Waitangi/Treaty of Waitangi obligations were addressed through the ethics process.

## Conflicts of Interest

Dr Wailling is an independent researcher and consultant who collaborates with healthcare, advocacy and academic institutions. Dr Wailling has experienced healthcare harm as a clinician and consumer. Mr Cameron is Ringatohu, Hauora Māori Tūmatanui, National Public Health Service. Dr Wailling and Mr Cameron co‐chair the National Collaborative for Restorative Initiatives in Health, Aotearoa New Zealand. Ms Berry is the founder of Mesh Down Under, established in 2012, which acts as an unpaid and voluntary consumer advocacy and online support group for consumers harmed by mesh. Ms Berry is a consumer representative on the Ministry of Health Mesh Round Table. Dr Middleton has no declarations.

## Supporting information


Supporting File

## Data Availability

Raw research data are not shared due to commitments made during the ethics process.

## Footnotes

1 The Unfortunate Experiment was the title of the Metro Magazine article that exposed unethical practices at National Women's Hospital in the 1980s. In response, the Cartwright inquiry 1988 led to the establishment of the Office of the Health and Disability Commissioner, who is responsible for upholding the Health and Disability Commissioner Act 1994 & Code of Health and Disability Services Consumers' Rights 1996 and overseeing the National Advocacy Service.
