# Supplementary material for: Humanising Government Inquiry: A Realist Examination of a Restorative Approach to Surgical Mesh Harm in Aotearoa New Zealand
Source: Health Expect. 2026 Oct 7;29(5):e70897. doi: 10.1111/hex.70897 (PMC13643949; doi:10.1111/hex.70897)
Supplement: Supplementary file 1 [file HEX-29-e70897-s001.docx]

**Supplementary file 1: All data sources**

| **Data source (Research phase)** | **Data included in the analysis (Publication date/data collection period)** | **Number** | **Identifier** |
| --- | --- | --- | --- |
| Public reports that were commissioned by the Office of the Chief Clinical Officers for the purpose of transparently reporting findings to the public (1) | 1. Data from the MOH 2018 survey that investigated how people affected by mesh wanted to share their experiences 2. Analysed data published in the programme report that detailed participants’ experiences of surgical mesh use (9) 3. Analysed quantitative data that was published in the programme report that detailed the descriptive evaluation | 3 | Reference and page number |
| Programme documents that detailed the co-design and operational delivery (1&2) | Meeting notes, programme protocols, public communications, operational documents (May 2019–December 2019) | 9 | PD (page number). |
| Qualitative survey data collected for the purposes of realist and descriptive evaluations (1&2) | Free-text survey comments from affected consumer respondents who participated in the programme (n=215) (June 2020-September 2020) | 215 | Consumer (number) e.g., C123 |
| Qualitative interview data collected for the purposes of realist and descriptive evaluations (2) | 13 hours and 32 minutes of interview data. (June 2020-September 2020) | 15 | Interview participant (number) e.g., IP3 |
| Qualitative focus group data collected for the purposes of testing and refining initial CMO configurations (3) | 1 hour and 29 minutes of interview data (December 2023)  Observation records. | 8 | Workshop participant (number) e.g., WP4 |
| Public documents and reports that relate to the work of the Collaborative (3) | He maungarongo ki ngā iwi: Envisioning a restorative health system in Aotearoa. | 1 | Reference and page number |
| Research diary (1-3) | Reflections, observations and the decision points captured during the conception, design, implementation, and evaluation of the programme and the research processes overall (May 2019–March 2024) | 1 | RD (page number) |
| Empirical and grey literature examined for the purpose of developing programme theories (1-3) | Empirical and grey literature sourced from academic, policy and non-traditional publishing sources (various) | Multiple sources | APA reference |

**Supplementary file 2: VICTORE Framework** Pawson R. The science of evaluation: A realist manifesto. London: SAGE; 2013. p.43

| **Component** | **Definition applied in theory testing** | **Realist evaluative purpose** |
| --- | --- | --- |
| Volitions | The choice architecture of the programme was examined to identify: (a) the programme resources offered (and removed) to (and from) the participants in order to enact the underlying principles, practices, and goals; and (b) the way in which programme participants engaged with and responded to programme resources | Mechanism development  To identify the outcomes participants desired |
| Implementation | The processes through which the programme was operationalised.  An examination of fidelity to the programme protocol and adaptations | To identify the characteristics of compounded harm and how the implementation approach influenced outcomes |
| Contexts | The infrastructural system (the wider social, economic and cultural factors); institutional settings (including the rules, norms and customs that surround them); the interpersonal relationships (which constitute the relational structure within which individuals are embedded); and the individual capacities of the key actors (Pawson 2006) | To identify contexts in which mechanisms fire (or not) |
| Time | The history of surgical use and the timing of the programme. The history of similar programmes that have attempted to resolve harm from surgical mesh use or healthcare harm more broadly | To identify the historical contexts into which the programme was introduced and how it may have influenced participants’ engagement choices and experiences |
| Outcomes | The wide range of outcomes the programme introduced | To identify demi-regularities and explain outcomes using the theory of generative causality |
| Rivalry | The potential impact of other programmes in the field | To map the pre-existing policy landscape and contexts into which the programme was embedded, how it influenced participants, and how it modified the delivery of the programme |
| Emergence | The combining of programme components to produce novel or unexpected outcomes | To identify the emergent effects, long-term adaptations, societal changes, and unintended consequences associated with the programme, and examine if the spread and duplication of the programme might blunt its effectiveness |

**Supplementary file 3: Theory testing and refinement data collection tools**

**Supplementary file 4: Theory development**
